# Portugal nautical stations: Strategic alliances for sport tourism and environmental sustainability

**DOI:** 10.3389/fspor.2022.982691

**Published:** 2022-09-12

**Authors:** Elsa Pereira, Rute Martins, João Filipe Marques, Adão Flores, Vahid Aghdash, Margarida Mascarenhas

**Affiliations:** ^1^School of Education and Communication, University of Algarve, Faro, Portugal; ^2^Research Centre for Tourism, Sustainability and Well-Being (CinTurs), University of Algarve, Faro, Portugal; ^3^Faculty of Economics, University of Algarve, Faro, Portugal; ^4^Faculty of Human Kinetics, University of Lisbon, Lisbon, Portugal

**Keywords:** nautical stations, strategy, sustainability, alliances, sport tourism

## Abstract

Nautical tourism is a tourist product with great development in the European space and a lot of potential to promote and develop tourist destinations. Considering the dynamics of nautical tourism management and the importance of meeting the specificities of this market niche, the objective of this study was to analyse the strategic alliances for the development of the offer of nautical tourism products, namely the strategic goals and sustainable environmental practices adopted by their actors which integrated the strategic alliances in order to certificate a plethora of nautical stations in Portugal. Between September and December 2021, 17 Portuguese nautical stations' application forms were collected. Content analysis using Nvivo software was the technique used for data analysis. The results showed a multiplicity of strategic objectives associated with the strategic alliance established between the nautical stations. The strategic vision of nautical stations for the development of strategic alliances is built, firstly, on the objective of structuring the tourism offer, followed by increasing governance and promoting and marketing nautical tourism using the image of the destination. Based on these results, it is possible to infer the importance of nautical stations in destination competitiveness and the role of strategic alliances in facilitating penetration in the nautical tourism market. The managers of nautical stations should consider the use of strategic alliances to make a cooperative marketing in order to improve the experience of the clients. Regarding environmental sustainability practices, the results exposed the prominence of environmental education actions in contrast to the reduced number of nautical stations developing actions for the adoption of sustainable transport. This study contributes to a better understanding of nautical tourism and Portuguese nautical stations, a project of strategic investment in sport and tourism, inferring on the objectives underlying the formation of strategic alliances and on the adopted environmental sustainability practices. The conclusions of this study point to the need for future scientific research on the actual operationalization of the objectives underlying the formation of strategic alliances, as well as the environmental practices developed by nautical stations.

## Introduction

The last decades of the twentieth century have observed the development of strategic alliances as the most significant change in the business context (Peroff et al., 2017). According to the authors, the establishment of strategic alliances is the only way by which organizations have attempted to respond to changes in the market, while simultaneously maintaining relationships with current customers and expanding their relationships with the main customers. The main objective of forming strategic alliances is to minimize risk, while maximizing market presence (Harbison and Pekar, 1998) and synergistically increase the organization's competitiveness, through access to external sources and promoting learning and rapid changes.

A strategic alliance is a long-standing relationship between two or more partners within a demand chain to improve and develop mutual agreement strategies in terms of common goals and contextual opportunities (Eisenhardt and Schoonhoven, 1996). Bitran et al. (2002) define a strategic alliance as a strategic agreement between two or more organizations who want to improve their competitive position and performance through shared resources.

The loss of identity and independence of corporations, as the result of strategic alliances, has become an obsolete idea. Hence, the creation of a strategic alliance between organizations requires considering the process of alliance adaptation and its drivers (Reuer and Zollo, 2000). In some national and international markets, strategic alliances have changed the underlying model of competition, from the traditional competition of the company to the company to compete against the network (Kotler and Keller, 2016). Strategic contributions differ in terms of the level of cooperation of partners and value (Larrinaga, 2017).

In general, the notion of strategic alliances is based on three principles (Masselink et al., 2016): (i) the partnership between partners is formal and informal; (ii) existence of at least two partners; and (iii) achieving strategic goals. Also, four types of strategic alliances can be introduced (Rodrigues, 2016): (a) joint venture—it is the most common type of unification, by which a business partnership activity is formed by two or more organizations with strategic objectives, generating independent institutions, and each of these entities allocates operational responsibilities, financial hazards and rewards while their independence and identity are maintained; (b) consortium of mutual services—it is the involvement of similar firms in industries that integrate their resources to obtain advanced benefits and technology, which otherwise, would be highly expensive to achieve; (c) licensing agreement—it is an agreement under which the exporting company grants a legal license to another company to produce commodities, and the receiver company pays a license to the issuing company. This alliance is useful when the business sign of the donor company is well-known; (iv) participation in the value chain—it is a strong and close union where a firm or business unit forms a long-term agreement with suppliers or key distributors to gain a competitive advantage.

Creating a strategic alliance has many benefits (Carayannis et al., 2000), such as: (a) scale savings and savings resulting from the scope; (b) quick and easy access to knowledge and market; (c) reducing the capital needs and the risks involved in the development of new products and technology; (d) effect of competition on relevant markets; (e) reduce the political and financial risk; (f) achieve a competitive advantage; (g) improvement of sales growth; (h) generating engagement in the business portfolio; and (i) increasing revenue. Several authors have stressed that in small and medium entreprises (SME) this business strategy is much more important (Kipley and Lewis, 2008; Zhao, 2014; Sefiani et al., 2018). The strategic alliances are also highlighted as beneficial in the production and in the service sectors, such as tourism (del Barrio-García and Prados-Peña, 2019) and sport tourism (Wäsche and Woll, 2013).

Tourism and sport are key elements of today's culture and have a special effect on social behavior. Sport is an important activity of tourists during tourism, and tourism and travel products/services are accompanied by different types of sports offers (Ito and Higham, 2020). Sport is a common motivation for tourists, highlighted by their tendency to participate in sports (Ito and Higham, 2020).

Sport tourism is interpreted as a leisure sport trip that temporarily pulls individuals out of their community (Gibson, 2006). In this way, it can cover trips away from home aiming to practicing sports and play, sport watching, visiting sports attractions, involving both competition and competition activities (Hudson, 2003). Therefore, it can be said that any type of travel for sport activities is called sport tourism and may take place individually or collectively (Luković, 2013).

The sport tourism product is a multi-dimensional combination of services and experience opportunities such as transport, lodging, sport activities and facilities, infrastructure, natural surroundings, and social contacts (Murphy et al., 2000; Tuppen, 2000; Thwaites and Chadwick, 2006). As the authors stressed, this kind of offer are provided by a vast array of actors in the visited region contributing to the sport tourism experience. Thus, the quality of the whole sport tourism experience is determined by the combination and coordination of a bundle of diverse services and goods provided by different stakeholders (actors) within the tourist region (Woods and Deeganm, 2006; Elbe et al., 2009). Furthermore, aspects of physical appearance such as beautiful landscapes, attractive and well-maintained areas and spaces for sport tourism, and the attitude of local residents toward sport tourism activities, are relevant. Since quality in sport tourism depends on of many different elements, the sport tourism product must be understood as the overall sport tourism experience as perceived by a visitor (Harrison-Hill and Chalip, 2005). Different social systems such as sport, economy, the systems of health, leisure, ecology, and politics, as well as several subfields with differing interests, create the sport tourism context and therefore a complicated field (Hinch and Higham, 2004). Wäsche and Woll (2010, 2013) have stressed that this inherent complexity results in a great number of actors from different sectors with different organizational cultures, interests, and goals. For the authors, public organizations (e.g., regional administration and infrastructures, tourist boards, and public sport spaces/facilities), non-profit organizations (e.g., sport clubs) as well as for-profit organizations (e.g., skiing schools and sport rentals) play an important part in contributing to a regional sport tourism product. Furthermore, hotels, retailers, farmers, local residents, and pressure groups (e.g., environmental protection bodies) must be considered. It is a key issue in sport tourism that requires nature-based resources and infrastructural arrangements which might have significant ecological and social impacts and subsequent problematic issues (Bull, 2005; Hall, 2005). In sum, the highly heterogeneous group of actors constitutes a specific feature of sport tourism and contributes to making the management of a sport tourism destination a complex task (Tuppen, 2000; Hall, 2005; Wäsche and Woll, 2010, 2013; Ziakas and Costa, 2011).

Fredline and Faulkner (2001) advocate that one of the most important ways by which sport tourism industry can improve global competitiveness is to create strategic alliances with other members of the industry. Concerning regional sport tourism development, the cooperation of a very diverse group of actors (individual or corporate) from different social systems is crucial. Specifically, the differing interests of various regional stakeholders in sport tourism have to be coordinated. Also, collective efforts are required to provide a sport tourism experience for visitors with a diverse range of products, aiming for a positive and sustainable regional development. Subsequently, a key challenge in managing regional sport tourism is the intersectoral integration of a heterogeneous group of actors (Tuppen, 2000) who act both as single actors, and simultaneously, as a collective actor in organizing and providing the overall sport tourism product of a region. However, there is only limited scientific knowledge about organizational structures, mechanisms, and processes in strategic alliances (Wäsche and Woll, 2010, 2013; Kennelly and Toohey, 2014). Hence, it is crucial to understand the complex interplay of single actors' actions and the development of collective structure through regional cooperation in sport tourism (Mollah et al., 2021).

For a deeper understanding of the phenomenon, and since sport tourism is highlighted by the World Tourism Organization (2019) for its potential to promote environmental sustainability, both by raising awareness and encouraging the adoption of pro-environmental measures, being one of the fastest growing tourism segments (Alexandris and Kaplanidou, 2014), it is important to understand this complex interplay also in an environmental sustainability perspective.

According to the European Commission (2014), coastal tourism is a large part of tourism, which employs more than 3.2 million people, produces more than one-third of the world's economy, a total of 183 billion euros. Around 51% of the EU accommodation capacity is concentrated in coastal areas (Weston et al., 2019).

Nautical tourism activity is a scattered industry based on small businesses, hampering managers' control of all components of the tourism system and/or all elements of the decision-making process (Goni and Yustika, 2019). Verdet (2002) places nautical tourism within the framework of a set of relationships between people who come together when they travel for less than a year and whose main motivation is to carry out nautical activities. Due to the multifaceted nature of tourism, new typologies have come into existence and many different forms of tourism have co-existed over the last decades, as water tourism (Jennings, 2007), lake tourism (Hall and Härkönen, 2006) and more recently, nautical tourism (Luković, 2013). Jennings (2007) advocates the concept of water-based tourism because it “relates to any touristic activity undertaken in or in relation to water resources, such as lakes, dams, canals, creeks, streams, rivers, waterways, marine coastal zones, seas, oceans and ice-associated areas” (p. 10). In this point of view, this form of tourism is strongly resource-based, i.e., the natural resource (water) firmly determines the whole development and activity (boating, sailing, surfing, fishing, 1-day tours, scuba diving, etc.). Luković (2013) defines nautical tourism as a sum of poly-functional activities and relations that are caused by the tourist stay within or out of the ports of nautical tourism, and by the use of vessels or other objects related to the nautical and tourist activities, for the purpose of recreation, sport, entertainment or other needs. In relation to the differences that may exist between nautical, maritime, and marine tourism (Forteza et al., 2017), there is no unanimity or clarity among the authors. In general terms, the differentiating element attributed to nautical tourism is the practice of sporting activities at sea (Carrasco, 2001; Luković, 2013) which can also be carried out in other aquatic environments (Jovanovic et al., 2013).

Nautical tourism is considered a recent commercial activity that has been developed between ordinary tourism and maritime activity, comprising characteristics that make it a special type of tourism (Kovačić et al., 2006). The authors point out the importance of developing a relatively new nautical market, defining it as a system that is divided into technological subsystems at sea and on land (Kasum et al., 2011). Nautical tourism is a diversified branch of general tourism that has significantly changed the structure and peculiarities of the tourism industry (Kovačić and Favro, 2012). These authors underline that nautical tourism is a variety of tourism with the sea as a distinctive element where the marinas are considered central facilities of nautical tourism, dedicated to satisfying the complex and growing demand of the nautical tourist (Benevolo and Spinelli, 2018). It is a complex system that uses various forms of technical and technological processes, hence it is exposed to certain risks (Kasum et al., 2018). Nautical tourism is a relevant category of maritime tourism, since it generates direct impacts on coastal development and destination promotion and has become one of the most important areas of research (Bal and Czalczynska-Podolska, 2019). As highlighted by Vázquez (2020), nautical tourism in the Mediterranean Sea is greatly dynamic and developed. Especially, for tourists from the cold North of Europe, the mild Mediterranean climate gives the opportunity to go on vacation almost all year round. Nevertheless, the summer season remains particularly popular, creating a strong seasonal character in nautical tourism. The European Atlantic coast nautical tourism is very well developed despite the climate, which is a consequence of the high degree of development of countries in this part of Europe (Masselink et al., 2016).

Nautical tourism is a highly dynamic product with great potential to develop consolidated destinations and can serve destinations that are not attractive for development (Javaloyes, 2012). The success of this type of tourism depends on the wide range of activities it offers and on the possibility of integrating it with active tourism and contact with nature (Perelló, 2013). However, due the the fragility of coastal ecosystems and landscapes, the European Union (EU) and numerous international organizations have concerned about the most appropriate approach for the development and the management of coastal zones.

Currently, the pursuit of the implementation of sustainability by society has a guiding political panel—the Sustainable Development Goals (SDG) within the United Nations Agenda 2030 (United Nations, 2015). The environmental pillar of sustainability, defined as “a condition of balance, resilience, and interconnectedness that allows human society to satisfy its needs while neither exceeding the capacity of its supporting ecosystems to continue to regenerate the services necessary to meet those needs nor by our actions diminishing biological diversity” (Morelli, 2011, p. 6), is contemplated by the SDG and has already been embraced by the main international organizations that lead sport (International Olympic Committee, 2012), and particularly, sport tourism (World Tourism Organization, 2019). The creation of the Sports for Climate Action Framework (United Nations Framework Convention on Climate Change, 2019a) is an example of the sport community's willingness to act on behalf of the environmental cause. This framework calls for the commitment of sport organizations to adopt strategies that aim and operationalize the climate action, spreading the environmental message within the sport community. Among the participating organizations, there are several acting in the coastal and maritime context (United Nations Framework Convention on Climate Change, 2019b), namely: (a) federations and leagues (e.g., World Surf League, CSA Surf Canada, World Sailing, World Rowing Federation, International and World Rafting Federations); (b) national teams (e.g., Sail GP team—United States, Great Britain, Australia, Japan, France; and (c) sporting events (e.g., The Ocean Race).

In the context of nautical tourism, the Fédération Européenne de Destinations Touristiques Nautiques prepared a declaration (Fédération Européenne de Destinations Touristiques Nautiques, 2012), exposing the sector's concern regarding the integration of sustainability, in which 10 objectives are proposed, highlighting: preservation of coastal ecosystems; protection of natural areas and endangered species in the exercise of nautical activities; reduction in the consumption of natural resources, waste and polluting products; promoting environmental education and awareness; innovation in the management and marketing of nautical products and services in order to promote environmental sustainability; and introduction of environmental criteria in the involved organizations' management policies. In this scenario, the Portuguese organization “Fórum Oceano: Associação da Economia do Mar,” an association that manages the Portuguese sea cluster, established the regulation for the certification of nautical stations that intend to integrate the network of Portuguese Nautical Stations, including a criterion for environmental sustainability, asking for “reference to actions to ensure the environmental sustainability of interventions” (Fórum Oceano, 2019, p. 10). Recognizing the importance of implementing and measuring environmental sustainability in nautical tourism is not only desirable, but absolutely necessary.

The environmental sustainability of sport tourism developed in the context of coastal and maritime areas has been investigated in order to understand the influence of the various factors that contribute to its implementation (Mascarenhas et al., 2021). For instances, a greater sporting experience of divers has been associated with a sport practice that is less harmful to the marine biota (Hammerton, 2017). Conversely, the use of accessories, such as cameras or musk sticks, has been associated with higher levels of destruction of the marine space used by snorkelers and divers for sporting purposes (Hammerton, 2017; Giglio et al., 2018). Several strategies have been advanced to mitigate these negative impacts on marine biota (Giglio et al., 2018), such as: zoning, i.e., limiting the access of tourists (or those with low sport practice experience) to more environmentally sensitive locations; and the promotion of good diving/snorkeling practices through short video-briefings. Additionally, resorting to the implementation of artificial reefs can satisfy the various segments of dive tourism, where tourists with less sporting experience can dive in a space suitable for their sport skills. These strategies promote the protection of fauna and flora in diving spaces, relieving the pressure of mass tourism in the natural space (Belhassen et al., 2017).

The environmental impact of sport tourism is not just a result of the pressure of recreation in the natural space. Unequivocally, it is also necessary to monitor and analyse the carbon emissions generated by the activities carried out in the context of sport tourism (Mascarenhas et al., 2021). The promotion of energy efficiency, consumption of environmentally friendly products and implementation of recycling/reuse programs by sport tourism operators are frequently cited examples of good environmental management practices by coastal tourism operators (Carneiro et al., 2016; Yfantidou et al., 2017). In conjunction with these environmental practices, the importance of the collaborative factor for the implementation of more environmental management has also been highly recommended (Mascarenhas et al., 2021). Examples of positive results for the environmental sustainability of nautical tourism support the recommendation for processes of collaboration and participation of the main stakeholders: in Aljezur, Portugal, a municipal charter for sustainable management was elaborated with the collaboration of the main stakeholders, taking into account the convergence of several indicators, including environmental indicators regarding the surfing activity and the necessary actions for a better environmental management of the surf tourism in the destination (Machado et al., 2018); in Villefranche-sur-Mer, on the French Riviera, the management of the local nautical station was reconverted to meet the objective of presenting a current and environmentally conscious nautical tourism offer. The objectives relating to the promotion of high environmental quality supported the design of strategies that included the increase in the offer of recreational and sporting activities that meet certain environmental requirements, such as aqua gym, nautical trails, all derivatives of windsurfing, paddle, sea triathlon, sport swimming on the high seas, pedal boats, water polo and scuba (Coglievina et al., 2016).

However, as has been widely emphasized, transport is the biggest contributor to the generation of carbon emissions in the tourism sector (Scott et al., 2016). For this reason, nautical tourism also has to implement strategies and practices capable of mitigating climate change, with a special focus on transport sustainability.

Within the management tools intended to facilitate the implementation of environmental sustainability in coastal tourism, some can be highlighted, namely those focusing on: (i) the need for coordinated collaboration between all stakeholders, in order to infer on converging and divergent topics, enhancing possible synergies between extractive, recreational and natural space conservation use, framed in the ecosystem concept (Biggs et al., 2016; Chen et al., 2016); and (ii) the identification of indicators for assessing sustainability (Drius et al., 2019; Coccossis and Koutsopoulou, 2020). For example, Drius et al. (2019) present a conceptual framework that addresses the management of the environmental impacts of nautical tourism by analyzing the trade-offs between environmental threats from coastal tourism and other human activities and coastal ecosystem services. In turn, the study developed by Coccossis and Koutsopoulou (2020) elaborated and applied a tool for measuring and monitoring sustainability at the local level (e.g., a nautical station). This tool integrates three types of indicators to assess sustainability in nautical tourism: core indicators (i.e., general indicators for sustainable coastal tourism); destination indicators (i.e., to access the unique characteristics of different tourism products, such as beach/maritime tourism, urban/cultural tourism, cruising, recreational boating and nature/ecotourism); and area-specific indicators (i.e., incorporating the crucial and specific aspects of each destination to monitor sustainability). To ensure the effectiveness of the operationalization of this tool, the importance of collaboration among key stakeholders in selecting and prioritizing indicators, and obtaining data, is also highly recommended (Coccossis and Koutsopoulou, 2020).

In addition to applying tools to monitor and analyse environmental indicators, another alternative for the promotion of environmental sustainability involves the inclusion of marketing strategies to enhance the message and environmental action of nautical stations, such as co-branding. As a marketing strategy, co-branding “in which two or more brands are presented simultaneously to the consumer as one product to create a sum of brand assets, that is greater than that of the individual brands” (Turan, 2021, p. 1), may allow a more effective connection between the environmental image of the nautical stations and the corresponding tourist offer, highlighting the fact that the brand image fit is one of the success factors of this strategy (Turan, 2021). In this regard, the study developed by Hsiao (2018) exposed the incongruity of the co-branding strategy in relation to the image of a low carbon island that was presented to tourists incorporating a recreational offer that included high carbon activities, namely motorized nautical activities, which culminated in a mismatch between the image of the island and its tourist offer.

In this sequence, to achieve mutual benefits (i.e., either for the environmental image for a nautical station, or for the implementation of the environmental sustainability of the different products and services of nautical tourism), the following recommendations must be observed: (i) enhancing the offer of more sustainable alternatives from the inventory of nautical sport activities; and (ii) operationalization and dissemination of more environmentally friendly choices in other areas of the tourism offer, namely, in terms of accommodation and transport (Hsiao, 2018).

According to the exposed, the focus of this research is to analyse the strategic alliances for the development of the offer of nautical tourism products, namely its strategic goals and sustainable environmental practices adopted by the actors which integrated the strategic alliance in order to certificate a plethora of nautical stations in Portugal.

## Materials and methods

According to the National Strategic Plan for Tourism 2027 (Turismo de Portugal I. P., 2017), Portugal has an excellent coastline for surfing, recognized worldwide, as well as for sport and nautical activities; vast marine biodiversity; and natural and infrastructural touristic conditions for cruises. Some of the lines of action of the National Strategic Plan for Tourism 2027 are based on the axis of valorization of the territory with the affirmation of tourism in the economy of the sea, namely: (i) Reinforcement of Portugal's position as a destination for nautical, sport and leisure activities associated with the sea, on the entire coast, and as an internationally recognized surfing destination; (ii) Dynamization and valorization of infrastructures, equipment and services to support nautical tourism, namely, ports, marinas and nautical centers; (iii) Nautical activities for enjoyment of the sea connected to diving, sailing, canoeing, observation of cetaceans and seabirds, fishing, sightseeing tours and beach activities that integrate sustainability in the nautical culture of the sea; (iv) Promotion of “routes of experiences” and tourist offers around the sea and nautical activities; (v) Coastal enhancement actions, including the requalification of marginal areas and the appreciation of beaches; (vi) Tourism projects including health tourism' projects associated with the therapeutic properties of the sea; and (vii) Appreciation of seafood associated with the Mediterranean diet (Turismo de Portugal I. P., 2017). The inclusion of nautical tourism as a strategic product for Portugal is essential for valuing the product, both in tourism and in sport (Morais de Brito and Cordeiro, 2020).

In line with this recognition, between 2014 and 2015, the Business Association of Portugal, in cooperation with the Fórum Oceano (FO), developed the project entitled Nautical Portugal (Fórum Oceano, 2019). The main goal of the project was to potentiate the development of a collective strategy to accelerate the structuring of the nautical sector in order to compete in the global market. The aim of FO was to create, promote and certify nautical stations in Portugal (Fórum Oceano, 2019). The Regulation for the Certification of Nautical Stations (NS) of Portugal (Morais de Brito and Cordeiro, 2020) states that nautical stations are, for the most part, coastal destinations and nautical tourism with an excellent opportunity to reorient some sun and beach tourism destinations. Alongside, there are conditions in the interior territories for the certification of NS, in stable water plans, namely rivers, lakes and reservoirs of dams. For potential visitors, the network offering, under the name of NS, guarantees the quality of the tourist product and the services provided, as well as information support and reservation of accommodation and services (Morais de Brito and Cordeiro, 2020).

The Portuguese nautical stations (PNS) is our case study as an organized network that contributes to the valorization of nautical resources present in the territory (for more information visit http://www.forumoceano.pt/index.php). This network includes nautical activities, facilities such as accommodation, restaurants, and other important services for attracting tourists. The main goal of the PNS is to create and add value to a diverse and integrated experience, based on a cooperation platform between players who offer an organized touristic product or service. The data collection was developed through the stablisment of a protocol with the FO. The FO streamlined the authorization process for nautical stations to allow us access to the NS application forms to obtain certification. Portugal has 29 nautical stations certified from north to south and from the coast to the inland waters, of which 17 allowed the research team to consult the official aplications forms. As such, in this study the official application forms of 17 certified Nautical Stations were collected and analyzed. For the purpose of the present research, two dimensions of the applications forms were analyzed, namely the strategic goals and the environmental practices described by the diferent partners of the network.The inductive content analysis was the method pursued related to the study of the mentioned dimensions. According to Bardin (1977) “the content analysis appears as a technique conjunction of communications' analysis that uses systematic procedures and description objectives from the message content” (p. 38). That is why it is an effective method to many areas in the social empirical sciences and often used in tourism research (Rejowski, 2010). The emergent references related to strategic goals and environmental pratices were coded in open concepts. Sistematically, comparison of concepts led to the definition of the key code followed by an axial and selective coding which in turn, allowed the definition of the subcategories in the two analyzed dimensions. This process was done based on intercoder reliability procedures between tree of the co-authors. The NVIVO software was used to the codification process in order to explore patterns related with the dimensions in study.

## Results and discussion

The results show the multiplicity of strategic objectives associated with the established strategic alliance related to sport, namely nautical sport (Table 1).

**Table 1:** Strategic goals per nautical station.

**Strategic goals**	**Nautical stations (*n*)**
Structure the offer	16
Increase governance	11
Promote and market destinations	11
Increase sustainability	8
Train human resources	7
Create and improve facilities	7
Organize events	6
Intensify service quality	5
Develop accessible and inclusive services	4
Others	2

The nautical stations as a strategic alliance confirm the Masselink et al. (2016) theory since the following assumptions are verified: (i) the partnership is formal and informal; (ii) the existence of at least two partners; and (iii) the achievement of strategic goals. In sport tourism industry, strategic alliances can improve global competitiveness and the cooperation of a very diverse group of actors (individual or corporate) from different social systems is crucial (Tuppen, 2000).

Nautical stations can be framed in the concept of tourism destination competitiveness with a particular focus on sport (Happ, 2021), as defined by the author as “a place's ability to optimize its attractiveness for residents and non-residents, to deliver high-quality, innovative, and attractive sports tourism services and to gain market shares in domestic and global marketplaces, while ensuring that the available resources supporting tourism are used efficiently and sustainably” (p. 67).

The data show the importance attributed by most nautical stations to the structuring of the offer as illustrated by several quotations, such as: “create an integrated strategy for the development of the nautical product, aggregating the offer, with the involvement of all sectors of activity directly connected and other complementary ones (NS5). Or:

*Structuring the tourist offer, in terms of nautical, entertainment activities, catering, accommodation and other services relevant to the attraction of tourists. For this purpose, it is important to create packages that are sufficiently attractive to customers in terms of the offer per se, quality, follow-up and its relationship with the price (NS1)*.

Or:

*Enhance the offer of nautical activities, in particular wakeboarding, water skiing, canoeing, rowing, stand-up-paddle, tourist fishing and nautical tours, namely through the creation of a network of partners, including operators of nautical activities, clubs and nautical sports centers, accommodation, restaurants and bars, and the main municipal and regional entities* (NS2).

The structuring of the offer is a fundamental strategic objective as the sport tourism product is a multi-dimensional combination of services and experience opportunities such as transport, lodging, sports activities and facilities, infrastructure, natural surroundings, and social contacts (Thwaites and Chadwick, 2006; Woods and Deeganm, 2006; Elbe et al., 2009). Some authors Zehrer et al. (2017), Aicher and Newland (2018), Newland and Aicher (2018), and Happ (2021) stressed that there are different types of experiences and different types of sport tourism consumers, such as active tourists and athletes; summer and winter sport tourists; different views in the range of stakeholders; new sports trend. For example, for active sport tourists, the quality of the sport experience and sport entertainment were vital (Aicher and Newland, 2018); on the contrary, for athletes, the event's reputation and status, constantly renewed event experience, and playing to the limit were more important (Getz and McConnell, 2011). On the whole, as there are different consumers with different interests, when working as a NS approach, the destination should offer different experiences, creat packages composed by different sports and atribbuts/atractions of the destinations and design specific offers for each target group (e.g., athletes or active).

Promoting and marketing destinations, as well as increasing governance, were also mentioned as strategic objectives by a large number of nautical stations (11 of the 17 NS). Regarding promotion, the role of NS in projecting the image of the destination as a nautical destination is mentioned (e.g., NS1; NS3; NS10; and NS14). As attested by the quotation of the NS1 “project the [] as a nautical destination in international markets, through a communication campaign aimed at specific target groups that have as aspiration the practice of nautical in articulation with the natural and cultural heritage,” or “promotion of the territory to increase the market share of visitors from abroad (mainly from Spain)” (NS14).

This objective falls within the meaning of Carayannis et al. (2000) when considering that strategic alliances are a quick and easy way of access to market. Wäsche et al. (2013) stress that sport tourism organizations should engage in cooperative marketing in order to improve the experience to the clients. Specifically, in regions “characterized by small businesses this ‘imperative for cooperation' is critical for successful marketing and management in tourism” (Wilkinson and March, 2008, p. 27). The Portugal nautical stations are a model of promotion on an international scale. The study by Lam-González et al. (2019) shows the relevance of internationalization in the context of nautical tourism to increase competitiveness for destinations.

With regard to governance, the importance of NS is highlighted to “encourage the articulation of promoting agents with public and private entities, creating partnerships that generate value in the development of nautical tourism” (NS16), or “establish with partners and associated nautical actors, a regular policy of internal and external communication” (NS17). Or, as added in another quotation:

*Implement a collaborative network between local public and private actors representing civil society that works as a discussion group for different themes associated with nautical and is an aggregator element, lobbying institutions and guardianship in order to influence facilitating policies and modes of action of the economic exercise of the tourist activity and in particular the nautical one (NS1)*.

Thus, a vision of governance was highlighted in this study to the extent that shared management—a collaborative network between local public and private actors that functions as a discussion and decision-making group—was understood as a strategic objective associated with the creation of the nautical stations. Klijn (2008) accentuates that the term governance appears associated with the purpose of improving coordination between related actors in solving society's problems. It is underlined by Emerson et al. (2012) that this concept integrates, in addition to public administration, stakeholders, civil society and the community, which is in line with the findings of this study, as stressed by the quotation “ensure greater access for local populations to nautical activities—involvement and access of populations, with special emphasis on school and competitive sports” (NS5). Governance acquires even more relevance insofar as, since tourism is considered as a complex system (Baggio et al., 2010), sport tourism, due to the intensification of the defined characteristics, can also be considered a complex system, implying that “the governance of a destination is controlled by a limited number of entities and is further confirmation of the necessity of creating cohesive inter-organizational networks for the production of integrated tourism experiences” (Baggio et al., 2010, p. 55). Moreover, the cooperation is considered crucial for the operative field of sport tourism management (Wäsche and Woll, 2013).

Sustainability, namely from a perspective associated with environmental protection and sustainable mobility, is a strategic objective for eight of the nautical stations under study. Quotes such as: “promotion of awareness-raising actions for the protection of the coastal area, promoting sustainable behaviors” (NS16), or “betting on sustainable development as a collective commitment, valuing and respecting the environment and territorial balance” (NS11) attest to the aspect of ecological sustainability. Moreover, “create sustainable dynamics of use, enhancement and preservation of the natural and environmental heritage linked to nautical activity, as well as the cultural and identity heritage of the region” (NS5).

Attending to cooperation as a determining factor for the implementation of a more environmental management in sport tourism (Mascarenhas et al., 2021), strategic alliances in sport and tourism should consider the strategic dimension related to the environmental sustainability (e.g., social and ecological compatibility: Wäsche et al., 2013). This dimension is particularly relevant since the nautical stations inscribe their action in natural ecosystems and the respective resources are key elements from the perspective of the sport tourist experience and have a powerful effect on the tourist perception of a chosen sport tourism destination (Hinch and Higham, 2004). As Perić et al. (2016) advocate, the environment is one of the vital elements in the key resources of the business model in sport tourism. Thus, it is important to integrate this dimension in product design, in the analysis of tourist flows and consequent carrying capacity, in the analysis of threats and enabling factors for tourism sustainability, littoralization and urbanization, land-sea interactions, coastal erosion and protection measures, water management, transport and accessibility, monitoring and measuring results.

The remaining strategic objectives—i.e., objectives focused on training human resources, creating and improving facilities, organizing events, intensifying service quality and developing accessible and inclusive services—were not mentioned in most of the analyzed forms, but worth to be mentioned as results of this study. As for example “NS will emphasize its strategy in training existing nautical operators in the surroundings, to attract new target groups” (NS8), or “promotion of nautical and safety training with nautical education institutions promoting a close relationship between the nautical sector, companies operating in the field and the school community” (NS16).

The importance of training of human resources and knowledge transfer, as pointed out in this study, are related to the strategic alliances. In this line, Ferreira and Franco (2020) showed that strategic alliances create important benefits on the human capital of the small and medium enterprises. As the authors point out, human capital is affected by strategic alliances and the relationships developed between SMEs and other companies are increasingly important for their growth. An isolated performance in the market can negatively influence the development capacity of this type of enterprises.

The creation and improvement of facilities/infrastructures was considered a priority insofar as many of the existing structures are geared toward a purely sporting and non-sports-tourist offer. As stressed in the quotation “dynamize and enhance the infrastructure, equipment and services to support nautical tourism” (NS10) or “creation of conditions and incentives for the requalification of existing spaces linked to nautical and/or implementation of new support structures” (NS12). In fact, Ivanić et al. (2018) refer to investment in infrastructure construction as an important factor in the development and enhancement of nautical tourism insofar as the existing facilities do not include the tourist vocation.

It should be noted that sporting events are referred to as strategic for attracting tourists, but also as a way to prolong the stay (NS1; NS10) in association with other events, as quoted:

*Promote, in partnership, a set of nautical events in complementarity with other entertainment events linked to the local culture and environment that offer the visitor the possibility of experiencing diversified experiences that contribute to increasing their stay in the territory (NS1)*.

The category “others” includes objectives with only one occurrence, namely, “capitalizing on knowledge networks and their transfer between territories” (NS1), as well as “creating normative elements for the development of nautical activities” (NS13).

The results of the practices of environment sustainability were categorized into five areas of action (Table 2), evidencing the concern of the NS coordinators for this world urgency.

**Table 2:** Environmental sustainability practices per nautical station.

**Environmental sustainability practices**	**Nautical stations (*n*)**
Environmental education	13
Resources management	8
Monitorization	7
Guidelines for environmental responsability	7
Sustainable transportation	3

The results lead to the conclusion that the environmental sustainability practices of the nautical stations follow the trend of implementing the environmental practices identified in the context of sport tourism, namely, the prevalence of environmental education and awareness actions (Mascarenhas et al., 2021) but little attention given to the implementation of the sustainable transport (Martins et al., 2021; Mascarenhas et al., 2021). Practices in terms of environmental education are mentioned by most of the EN in the study, namely through “awareness actions on sustainable tourism and environmental protection” both for the network partners and for local communities, as well as for the customers of the NS (e.g., NS2, NS4, NS6, NS7, and NS14), namely: “awareness-raising actions on sustainable tourism and environmental protection” (NS2); “awareness-raising and information activities, dedicated in particular to these issues (among which the effects of climate change, the presence of plastics in the oceans and the threat of protected species gain increasing expression” (NS4); “environmental awareness actions among network partners” (NS6); “creation of the Environmental Guide for sailors, whose objectives are: to improve environmental quality, safety, educate the youngest and participate in the preservation of natural resources” (NS7); and “develop and promote educational programs that encourage good practices in schools” (NS14).

As the results showed, the environmental education practices integrate several levels and involve different types of actors, such as children, providers and sport tourists. This way, by one side, it is important a high dregee of training of the managers in the environmental items, as they influence several actors and practices; by the other side, the providers in sport tourism must be informed about sport tourists behaviors in order to create clear explanations about the activities and their rules so negative impacts may be minimized (Perić et al., 2016) and benefits may be maximized whenever it is possible (min-max approach). It is also important to raise the awareness of the actors involved in the offer of nautical tourism to the need to create more benign alternatives for the environment, and to communicate and promote them in an effective and persuasive way, anticipating the factors that will influence their adoption by sport tourism consumers (Martins et al., 2021; Mascarenhas et al., 2021). Nautical stations are based in water/outdoor sports, mainly helded in natural spaces, and therefore, consumers and providers must be aware of the variables that can damage the environment. There are several examples of the negative impact of sport tourists activities and equipments on aquatic resources, namely, the erosion of marine biota caused by divers (Hammerton, 2017; Giglio et al., 2018) and the various types of pollution produced by recreational boat engines (e.g., noise, light pollution, oil discharges, and other waste: Perić et al., 2016). Nevertheless, water sports as, for example, sailing, rowing, canoying, surf, and other less polluting sports, could be inspiring to create a more environmentally friendly human-nature relationship.

Within the scope of resource management, practices focus on the management of water, beaches, nautical centers, marinas and other nautical support infrastructure as well as recyclable waste (NS8, NS9, NS11, and NS16). There is a NS that presents a management plan with the responsibility of each actor by area of intervention (NS17):

*Partner [X] undertakes to inspect all those involved in the events of good practices for the preservation of the aquatic environment, at the request of the partners responsible for them; as well as inform the authorities of any anomaly detected, either in the aquatic environment or on its banks. Partner [Y] undertakes to carry out at least one environmental awareness campaign in schools. Partner [Z] is committed to cleaning the space surrounding the water access platform and raising awareness of the adoption of behaviours consistent with protecting the environment, such as not throwing debris on the floor, using reusable bottles/drums and not voluntarily expel secretions into the surrounding environment*.

Monitoring is referred to at several levels: (i) number of visitors and their impacts/carrying capacities (NS4, NS10); (ii) waste (NS4); (iii) waters (NS9, NS10, NS11, NS15); (iv) biodiversity (NS11, NS15); and (v) consumption (energy and water) (NS11). For example:

*The environmental and territorial enhancement and qualification of the Municipality is supported by an integrative, dynamic and technologically advanced management model that allows the permanent availability of information on the various environmental components, thus contributing to the permanent and objective monitoring of the consequences associated with taking decision. On the coast, there is monitoring of bathing water quality, and biodiversity monitoring is also carried out (NS15)*.

In the application forms of the nautical stations, the adoption of guidelines for environmental responsibility was pointed out: (i) already created by other entities (e.g., European Charter for Sport and Sustainable Tourism; Code of Conduct and Good Practices of Portuguese Geoparks); (ii) created (NS1) or being created (NS2, NS4), within the scope of the nautical station [e.g., “in the process of adapting various models/regulations and existing manuals, to create a specific behavior manual, for the station [NS name omission] or the [NS name omission], which covers partners in the five municipalities, has a quality benchmark in preparation” (NS4)].

Resources management, monitoring and guidelines for environmental responsibility should be incorporated into practices associated with strategic management of the environment. Coccossis and Koutsopoulou (2020) elaborated and applied a tool with three types of indicators to assess sustainability in nautical tourism: core indicators (i.e., general indicators for sustainable coastal tourism); destination indicators (i.e., to access the unique characteristics of different tourism products, such as beach/maritime tourism, urban/cultural tourism, cruising, recreational boating, and nature/ecotourism); and area-specific indicators (i.e., incorporating the crucial and specific aspects of each destination to monitor sustainability).

It was found that there are nautical stations with holistic solutions, which could constitute cases to be analyzed in more depth in order to replicate in other nautical stations. For example, NS15 has an Environmental Management and Information System, within the scope of the Municipal Geographic Information System, which constitutes an online platform that integrates information on various environmental descriptors: Water, Air, Biodiversity, Energy, Soils and Landscape, Waste, Noise and Environmental Education. As advocated by Carneiro et al. (2016) and Yfantidou et al. (2017), this type of measures lead to environmental savings, e.g., the promotion of energy efficiency, consumption of environmentally friendly products and implementation of recycling/reuse programs, and are developed by sport tourism operators. Perić et al. (2016) advocate that “one of the possible solutions for reducing negative effects on the environment is fostering eco-innovations, a technological term usually closely correlated to eco-efficiency and ecological design”. Kelly et al. (2007) have found that significant tourist support existed for options that could increase the overall eco-efficiency of destinations. The study devoloped by Trstenjak et al. (2020) on nautical tourism in the Mediterranean shows that it is important to have more information about the creation of more environmentally friendly processes as it concludes that there are three major obstacles to greater renewable energy sources: “a lack of awareness and knowledge related to available EU funds intended for achieving sustainable business models and products, attractive financing opportunities for sustainable projects, and complicated bureaucratic procedures” (p. 12).

The area of sustainable transport is contemplated through the implementation of more ecological modes of mobility, such as electric vehicles and bicycles, the evaluation of the various flows related to demand and feasibility studies regarding the implementation of public transport (NS5, NS6, NS11). As pointed out in these quotations: “implementation of sustainable mobility measures” (NS5); “smooth modes of mobility such as electric vehicles and bicycles are available on the routes” (EN6); “assessment of demand-related road flows and feasibility studies for the implementation of public transport” (EN11).

The development of alternatives to include sustainable transport is in fact a measure that must be considered in a context where natural resources are the key to tourist attractiveness. In this sense, Hsiao (2018) has already recommended for the implementation of the environmental sustainability of the different products and services of nautical tourism, the operationalization and dissemination of more environmentally friendly choices in other areas of the tourism offer, namely, transport. The specification of sport services, taking into account the characteristics of consumers for the adoption of more sustainable behaviors, is important for the implementation of more ecological services, such as the inclusion of sustainable transport alternatives (Martins et al., 2021; Mascarenhas et al., 2021). In this sense, it is of great importance to promote these services given the fact that sport consumers are influenced by a greater aesthetic need and a stronger connection to the local community when considering using sustainable transport in the context of sport tourism (Martins et al., 2021).

## Conclusion

To conclude, this study analyzed the strategic alliances for the development of the offer of nautical tourism products, allowing for a first overview of a pioneering project in Portugal of strategic investment in sport and tourism. The main strategic goal with these alliances was to structure the nautical tourism offer, as well as to increase governance and to promote and market destinations. Although in a less pronounced way, a concern to integrate sustainability in its ecological aspect also emerged as a strategic goal. The results of the adopted environmental sustainability practices showed that there are nautical stations with holistic solutions, which could constitute cases to be analyzed in more depth in order to replicate in other nautical stations, namely, the environmental education practices.

As PNS is a pioneering project and the structuration of the nautical tourism offer is a new area of sport management in Portugal. FO and managers of the nautical stations should create workshops and workgroups to training and to promote the discussion of solutions, sharing of knowledge and development of such offer.

Bearing in mind the urgency of climate action, it is important to raise awareness of FO for the importance of all nautical stations prioritize the integration of sustainability as a strategic goal; future studies should focus on case studies on nautical stations that develop alternatives for sustainable transport. In this way, it will be possible to understand the context of the implementation of this type of actions, as well as the different factors that facilitate and constrain their effectiveness.

Future research should try to analyse all the PNS and also the remaining dimensions of the applications forms 1. In accordance with the objectives of the study, the direct observation of the environmental sustainability practices implemented by the nautical stations was not carried out, which constitutes a limitation of this study. Thus, future studies should carry out the scientific follow-up of the operationalization of the strategic objectives and practices exposed by the entities, as well as the respective impacts on the management of nautical stations in order to create scientific knowledge for the management of sport tourism, particularly, the nautical tourism.

## Data availability statement

The original contributions presented in the study are included in the article/supplementary material, further inquiries can be directed to the corresponding author.

## Author contributions

EP, AF, JM, VA, and MM contributed to conception and design of the study. VA organized the database. EP, VA, and AF performed the analysis. EP and VA wrote the first draft of the manuscript. EP, AF, JM, RM, VA, and MM wrote sections of the manuscript. All authors contributed to manuscript revision, read, and approved the submitted version.

## Funding

This work was supported by National Funds provided by FCT—Foundation for Science and Technology under the project UIDB/04020/2020.

## Conflict of interest

The authors declare that the research was conducted in the absence of any commercial or financial relationships that could be construed as a potential conflict of interest.

## Publisher's note

All claims expressed in this article are solely those of the authors and do not necessarily represent those of their affiliated organizations, or those of the publisher, the editors and the reviewers. Any product that may be evaluated in this article, or claim that may be made by its manufacturer, is not guaranteed or endorsed by the publisher.

## References

[B1] Aicher T. J. Newland B. L. (2018). To explore or race? Examining endurance athletes' destination event choices. J. Vacat. Mark. 24, 340–354. 10.1177/1356766717736364

[B2] Alexandris K. Kaplanidou K. (2014). Marketing sport event tourism: sport tourist behaviors and destination provisions. Sport Mark Q. 23, 125–126.

[B3] Baggio R. Scott N. Cooper C. (2010). Improving tourism destination governance: a complexity science approach. Pechlaner H, editor. Tour Rev. 65, 51–60. 10.1108/16605371011093863

[B4] Bal W. Czalczynska-Podolska M. (2019). Landscape and cultural aspects of the coastal area of western pomerania as factors of development of maritime and nautical tourism. Identification and definition of conditions. IOP Conf. Ser. Mater. Sci. Eng. 471, 2034. 10.1088/1757-899X/471/10/102034

[B5] Bardin L. (1977). Análise de conteúdo. Lisboa: Edições, 70.

[B6] Belhassen Y. Rousseau M. Tynyakov J. Shashar N. (2017). Evaluating the attractiveness and effectiveness of artificial coral reefs as a recreational ecosystem service. J. Environ. Manage. 203, 448–456. 10.1016/j.jenvman.2017.08.020 28837911

[B7] Benevolo C. Spinelli R. (2018). The quality of web communication by Italian tourist ports. Tourism. 66, 52–62.

[B8] Biggs D. Amar F. Valdebenito A. Gelcich S. (2016). Potential synergies between nature-based tourism and sustainable use of marine resources: insights from dive tourism in territorial user rights for fisheries in Chile. Romanach S editor. PLoS ONE. 11, e0148862. 10.1371/journal.pone.0148862 PMC4811548 27023451

[B9] Bitran I. Bitran J. Conn S. Nagel A. Nicholls H. (2002). SMART: system for the development, management and support of strategic alliances. Int. J. Prod. Econ. 80, 3–10. 10.1016/S0925-5273(02)00238-4

[B10] Bull C. (2005). Sport tourism destination resource analysis, in Sport Tourism Destinations: Issues, Opportunities and Analysis, ed Higham J. (London: Routledge), 25–38.

[B11] Carayannis E. G. Kassicieh S. K. Radosevich R. (2000). Strategic alliances as a source of early-stage seed capital in new technology-based firms. Technovation. 20, 603–615. 10.1016/S0166-4972(99)00161-3

[B12] Carneiro M. Breda Z. Cordeiro C. (2016). Sports tourism development and destination sustainability: the case of the coastal area of the Aveiro region, Portugal. J. Sport Tour. 20, 305–334. 10.1080/14775085.2016.1220863

[B13] Carrasco S. (2001). La relevancia del turismo náutico en la oferta turística. Cuad. Tur. 7, 67–80.

[B14] Chen T. C. Ku K. C. Chen C. S. (2016). Collaborative adaptive management for bigfin squid applied to tourism-related activities in coastal waters of Northeast Taiwan. Ocean Coast Manag. 119, 208–216. 10.1016/j.ocecoaman.2015.10.010

[B15] Coccossis H. Koutsopoulou A. (2020). Measuring and monitoring sustainability of coastal tourism destinationsin the Mediterranean. Tourism. 68, 482–498. 10.37741/t.68.4.8

[B16] Coglievina C. Massiera B. Gonzales V. Mahmoud I. (2016). Territorial dynamics of the Bay of Villefranche-sur-Mer (French Riviera): from commercial and military vocation to tourism and sporting activities. An Bras Estudo Tur. 6, 76–84.

[B17] del Barrio-García S. Prados-Peña M. (2019). Do brand authenticity and brand credibility facilitate brand equity? The case of heritage destination brand extension. J. Destin. Mark. Manag. 13, 10–23. 10.1016/j.jdmm.2019.05.002

[B18] Drius M. Bongiorni L. Depellegrin D. Menegon S. Pugnetti A. Stifter S.. (2019). Tackling challenges for Mediterranean sustainable coastal tourism: an ecosystem service perspective. Sci. Total Environ. 652, 1302–1317. 10.1016/j.scitotenv.2018.10.121 30586816

[B19] Eisenhardt K. Schoonhoven C. (1996). Resource-based view of strategic alliance formation: strategic and social effects in entrepreneurial firms. Organ Sci. 7, 136–150. 10.1287/orsc.7.2.136

[B20] Elbe J. Hallén L. Axelsson B. (2009). The destination-management organisation and the integrative destination-marketing process. Int. J. Tour. Res. 11, 283–296. 10.1002/jtr.695

[B21] Emerson K. Nabatchi T. Balogh S. (2012). An integrative framework for collaborative governance. J. Public Adm. Res. Theory. 22, 1–29. 10.1093/jopart/mur011

[B22] European Commission (2014). A European Strategy for More Growth and Jobs in Coastal and Maritime Tourism. Available online at: https://eur-lex.europa.eu/legal-content/EN/TXT/?uri=CELEX:52014DC0086 (accessed June 4, 2021).

[B23] Fédération Européenne de Destinations Touristiques Nautiques (2012). European Manifest for Sustainable Nautical Tourism. Available online at: http://www.nautical-tourism.eu/upload/files/06_15%20Manifeste%20ANG%2012p(1).pdf (accessed June 5, 2021).

[B24] Ferreira A. Franco M. (2020). The influence of strategic alliances on human capital development: a study applied to technology-based SMEs. EuroMed J. Bus. 15, 65–85. 10.1108/EMJB-04-2019-0052

[B25] Forteza J. González Y. Ledesma J. (2017). Motivación, satisfacción e intenciones del turista náutico en la Ruta del Spondylus (Ecuador). Estud Perspect En Tur. 26, 267–285.

[B26] Fórum Oceano (2019). Regulamento para a Certificação de Estações Náuticas de Portugal. Available online at: http://www.forumoceano.pt/files/Regulamento%20ENP_2019.pdf?d=b03x (accessed June 5, 2021).

[B27] Fredline E. Faulkner B. (2001). Residents' reactions to the staging of major motorsport events within their communities: a cluster analysis. Event Manag. 7, 103–114. 10.3727/152599501108751515

[B28] Getz D. McConnell A. (2011). Serious sport tourism and event travel careers. J. Sport Manag. 25, 326–338. 10.1123/jsm.25.4.326

[B29] Gibson H. (2006). Leisure and later life: past, present and future. Leis Stud. 25, 397–401. 10.1080/02614360600896437

[B30] Giglio V. Luiz O. Chadwick N. Ferreira C. (2018). Using an educational video-briefing to mitigate the ecological impacts of scuba diving. J. Sustain. Tour. 26, 782–797. 10.1080/09669582.2017.1408636

[B31] Goni J. Yustika B. (2019). The presence of global value chain in coastal marine tourism. J. Ris. Manaj. Dan. Bisnis. JRMB Fak Ekon UNIAT. 4, 137–152. 10.36226/jrmb.v4i1.248

[B32] Hall C. (2005). Tourism: Rethinking the Social Science of Mobility. Harlow: Prentice Hall.

[B33] Hall C. Härkönen T. (2006). Lake Tourism: An Integrated Approach to Lacustrine Tourism Systems. Clevedon; Buffalo: Channel View Publications, 235. (Aspects of tourism).

[B34] Hammerton Z. (2017). Determining the variables that influence SCUBA diving impacts in eastern Australian marine parks. Ocean Coast. Manag. 142, 209–217. 10.1016/j.ocecoaman.2017.03.030

[B35] Happ E. (2021). Tourism destination competitiveness with a particular focus on sport: the current state and a glance into the future – a systematic literature analysis. J. Sport Tour. 25, 66–82. 10.1080/14775085.2021.1888775

[B36] Harbison J. Pekar P. (1998). Smart Alliances: A Practical Guide to Repeatable Success. San Francisco, CA: Jossey-Bass. 79–95.

[B37] Harrison-Hill T. Chalip L. (2005). Marketing sport tourism: creating synergy between sport and destination. Sport Soc. 8, 302–320. 10.1080/17430430500102150

[B38] Hinch T. Higham J. E. S. (2004). Sport Tourism Development. Clevedon; Buffalo: Channel View Publications, 254 (Aspects of tourism).

[B39] Hsiao T. Y. A. (2018). study of the effects of co-branding between low-carbon islands and recreational activities. Curr. Issues Tour. 21, 529–546. 10.1080/13683500.2015.1093466

[B40] Hudson S. (ed.). (2003). Sport and Adventure Tourism. New York, NY: Haworth Hospitality Press, 324.

[B41] International Olympic Committee (2012). Sustainability Through Sport-Implementing the Olympic Movement's Agenda 21. Available online at: https://stillmed.olympic.org/Documents/Commissions_PDFfiles/SportAndEnvironment/Sustainability_Through_Sport.pdf (accessed January 10, 2022).

[B42] Ito E. Higham J. (2020). Supplemental tourism activities: a conceptual framework to maximise sport tourism benefits and opportunities. J. Sport Tour. 24, 269–284. 10.1080/14775085.2020.1850322

[B43] Ivanić K. Perić HadŽić A. Mohović D. (2018). Nautical tourism: generator of croatian economy development. Pomorstvo. 32, 59–66. 10.31217/p.32.1.7

[B44] Javaloyes E. (2012). La gestión de instalaciones náuticas de recreo: su relación con el turismo náutico en la Costa Blanca. Rev. Investig. Tur. 4, 119–131. 10.14198/INTURI2012.4.06

[B45] Jennings G. (2007). Water-Based Tourism, Sport, Leisure, and Recreation Experiences. Oxford, NY: Elsevier.

[B46] Jovanovic T. Dragin A. Armenski T. Pavic D. Davidovic N. (2013). What demotivates the tourist? Constraining factors of nautical tourism. J. Travel. Tour. Mark. 30, 858–872. 10.1080/10548408.2013.835679

[B47] Kasum J. Mikuličić J. Z. Kolić V. (2018). Safety issues, security and risk management in nautical tourism. Trans. Marit. Sci. 7, 184–188. 10.7225/toms.v07.n02.008

[B48] Kasum J. Žani J. BoŽi K. (2011). Nautical Tourism. London: WIT Press. 597–602.

[B49] Kelly J. Haider W. Williams P. W. Englund K. (2007). Stated preferences of tourists for eco-efficient destination planning options. Tour. Manag. 28, 377–390. 10.1016/j.tourman.2006.04.015

[B50] Kennelly M. Toohey K. (2014). Strategic alliances in sport tourism: national sport organisations and sport tour operators. Sport Manag. Rev. 17, 407–418. 10.1016/j.smr.2014.01.001

[B51] Kipley D. Lewis A. (2008). The scalability of H. Igor Ansoff's strategic management principles for small and medium sized firms. J. Manag Res. 1, 1–26. 10.5296/jmr.v1i1.33

[B52] Klijn E. H. (2008). Governance and Governance networks in Europe: an assessment of ten years of research on the theme. Public Manag Rev. 10, 505–525. 10.1080/14719030802263954

[B53] Kotler P. Keller K. (2016). Marketing Management. 15 Edn. Boston: Pearson, 692.

[B54] Kovačić M. Bošković D. Favro S. (2006). Possibilities and limitations of spatial technical and technological development of a port of nautical tourism. Nase More. 53, 54–62.

[B55] Kovačić M. Favro S. (2012). Development possibilities of nautical tourism within the Zadar county. Pomorstvo. 26, 151–164.

[B56] Lam-González Y. León C. León J. (2019). Assessing the effects of the climatic satisfaction on nautical tourists' on-site activities and expenditure decisions. J. Destin. Mark Manag. 14, 100372. 10.1016/j.jdmm.2019.100372

[B57] Larrinaga O. (2017). Is it desirable, necessary and possible to perform research using case studies? Cuad. Gest. 17, 147–171. 10.5295/cdg.140516ov11538748

[B58] Luković T. (2013). Nautical Tourism. Boston: CAB International.

[B59] Machado V. Carrasco P. Contreiras J. P. Duarte A. P. Gouveia D. (2018). Governing locally for sustainability: public and private organizations' perspective in surf tourism at Aljezur, Costa Vicentina, Portugal. Tour. Plan Dev. 15, 692–704. 10.1080/21568316.2017.1415958

[B60] Martins R. Pereira E. Rosado A. Marôco J. McCullough B. Mascarenhas M.. (2021). Understanding spectator sustainable transportation intentions in international sport tourism events. J. Sustain. Tour. 1–20. 10.1080/09669582.2021.1991936

[B61] Mascarenhas M. Pereira E. Rosado A. Martins R. (2021). How has science highlighted sports tourism in recent investigation on sports' environmental sustainability? A systematic review. J. Sport Tour. 25, 42–65. 10.1080/14775085.2021.1883461

[B62] Masselink G. Castelle B. Scott T. Dodet G. Suanez S. Jackson D.. (2016). Extreme wave activity during 2013/2014 winter and morphological impacts along the Atlantic coast of Europe. Geophys. Res. Lett. 43, 2135–2143. 10.1002/2015GL067492

[B63] Mollah M. Cuskelly G. Hill B. (2021). Sport tourism collaboration: a systematic quantitative literature review. J. Sport Tour. 25, 3–25. 10.1080/14775085.2021.1877563

[B64] Morais de Brito M. Cordeiro A. S. (2020). Nautical stations: catalysts for sustainable tourism development – the case of the Sines Nautical Station, in Advances in Hospitality, Tourism, and the Services Industry, eds M. Morais de Brito, A. Dias, M. Patuleia (IGI Global), 171–86. Available opnline at: http://services.igi-global.com/resolvedoi/resolve.aspx?doi=10, 4018./978-1-7998-1522-8.ch010 10.4018/978-1-7998-1522-8.ch010 (accesed June 17, 2022).

[B65] Morelli J. (2011). Environmental sustainability: a definition for environmental professionals. J. Environ. Sustain. 1, 1–10. 10.14448/jes.01.0002

[B66] Murphy P. Pritchard M. P. Smith B. (2000). The destination product and its impact on traveller perceptions. Tour Manag. 21, 43–52. 10.1016/S0261-5177(99)00080-1

[B67] Newland B. L. Aicher T. J. (2018). Exploring sport participants' event and destination choices. J. Sport Tour. 22, 131–149. 10.1080/14775085.2018.1436464

[B68] Perelló J. (2013). Estudio Preliminar del Turismo Náutico. Cuba: La Habana.

[B69] Perić M. Vitezić V. Mekinc J. (2016). Conceptualising innovative business models for sustainable sport tourism. Int. J. Sustain Dev. Plan. 11, 469–482. 10.2495/SDP-V11-N3-469-482

[B70] Peroff D. Deason G. Seekamp E. Iyengar J. (2017). Integrating frameworks for evaluating tourism partnerships: an exploration of success within the life cycle of a collaborative ecotourism development effort. J. Outdoor Recreat Tour. 17, 100–111. 10.1016/j.jort.2016.10.001

[B71] Rejowski M. (2010). Produção científica em Turismo: análise de estudos referenciais no exterior e no Brasil. Rev. Tur. Em Análise. 21, 224. 10.11606/issn.1984-4867.v21i2p224-246

[B72] Reuer J. Zollo M. (2000). Managing governance adaptations in strategic alliances. Eur. Manag. J. 18, 164–172. 10.1016/S0263-2373(99)00088-2

[B73] Rodrigues A. (2016). Green Destination Image as a Promotional Tool for Developing Water-Based Destinations: Some Insights. Available online at: http://rgdoi.net/1013140./RG.2.2.16525.33768 (accessed June 17, 2022).

[B74] Scott D. Hall C. M. Gössling S. A. (2016). A report on the Paris Climate Change Agreement and its implications for tourism: why we will always have Paris. J. Sustain. Tour. 24, 933–948. 10.1080/09669582.2016.1187623

[B75] Sefiani Y. Davies B. Bown R. Kite N. (2018). Performance of SMEs in Tangier: the interface of networking and *wasta*. EuroMed J. Bus. 13, 20–43. 10.1108/EMJB-06-2016-0016

[B76] Thwaites D. Chadwick S. (2006). Service quality perspectives in sport tourism, in Sport Tourism: Concepts and Theories, ed Gibson H. J. (London: Routledge) (Sport in the global society).

[B77] Trstenjak A. Žiković S. Mansour H. (2020). Making nautical tourism greener in the mediterranean. Sustainability. 12, 6693. 10.3390/su12166693

[B78] Tuppen J. (2000). The restructuring of winter sports resorts in the French Alps: problems, processes and policies. Int. J. Tour Res. 2, 327–44. 10.1002/1522-1970(200009/10) 2:53.3.CO;2-P

[B79] Turan C. (2021). Success drivers of co-branding: a meta-analysis. Int. J. Consum. Stud. 45, 911–936. 10.1111/ijcs.12682

[B80] Turismo de Portugal I. P. (2017). Estratégia Turismo 2027. Available online at: http://www.turismodeportugal.pt/SiteCollectionDocuments/estrategia/estrategia-turismo-2027.pdf (accessed June 3, 2021).

[B81] United Nations (2015). Transforming Our World: the 2030. Agenda for Sustainable Development. Report No.: A/RES/70/1. Available online at: https://www.un.org/ga/search/view_doc.asp?symbol=A/RES/70/1andLang=E (accessed June 10, 2021).

[B82] United Nations Framework Convention on Climate Change (2019a). Sports for Climate Action Framework. Available online at: https://unfccc.int/sites/default/files/resource/Sports_for_Climate_Action_Declaration_and_Framework.pdf (accessed January 8, 2022).

[B83] United Nations Framework Convention on Climate Change (2019b). Participants in the Sports for Climate Action Framework. Available online at: https://unfccc.int/climate-action/sectoral-engagement/sports-for-climate-action/participants-in-the-sports-for-climate-action-framework#eq-1 (accessed January 8, 2022).

[B84] Vázquez R. (2020). Nautical tourism: a bibliometric analysis. J. Spat. Organ Dyn. 8, 320–330.

[B85] Verdet A. (2002). Puertos Deportivos: Repercusión de su Administración y Gestión en el Desarrollo del Turismo Náutico. Estrategias para el caso de la Costa del Sol. Tesis doctoral, Departamento de Economía y Administración de Empresas.

[B86] Wäsche H. Dickson G. Woll A. (2013). Quality in regional sports tourism: a network approach to strategic quality management. J. Sport Tour. 18, 81–97. 10.1080/14775085.2013.826593

[B87] Wäsche H. Woll A. (2010). Regional sports tourism networks: a conceptual framework. J. Sport Tour. 15, 191–214. 10.1080/14775085.2010.513146

[B88] Wäsche H. Woll A. (2013). Managing regional sports tourism networks: a network perspective. Eur. Sport Manag. Q. 13, 404–427. 10.1080/16184742.2013.811608

[B89] Weston R. Guia J. Mihalič T. Prats L. Blasco D. Ferrer-Roca N.. (2019). Research for TRAN Committee – European tourism: recent developments and future challenges. Brussels: European Parliament, Policy Department for Structural and Cohesion Policies.

[B90] Wilkinson N. March R. (2008). Conceptual tools for evaluating tourism partnerships, *in Network Analysis and Tourism: From Theory to Practice*, eds N. Scott, R. Baggio, C. Cooper (Clevedon; Buffalo, NY: Channel View Publications). 27–9. Available online at: http://site.ebrary.com/id/10257270 10.21832/9781845410896-006 (accessed June 17, 2022).

[B91] Woods M. Deeganm J. (2006). The fuchsia destination quality brand: low on quality assurance, high on knowledge sharing. J. Qual. Assur. Hosp. Tour. 7, 75–98. 10.1300/J162v07n01_05

[B92] World Tourism Organization (2019). Sport Tourism and Sustainable Development Goals. Available online at: https://webunwto.s3.eu-west-1.amazonaws.com/s3fs-public/2019-09/sporttourismandsdgs.pdf (accessed January 20, 2022).

[B93] Yfantidou G. Spyridopoulou E. Kouthouris C. Balaska P. Matarazzo M. Costa G.. (2017). The future of sustainable tourism development for the Greek enterprises that provide sport tourism. Tour. Econ. 23, 1155–1162. 10.1177/1354816616686415

[B94] Zehrer A. Smeral E. Hallmann K. (2017). Destination competitiveness—a comparison of subjective and objective indicators for winter sports areas. J. Travel. Res. 56, 55–66. 10.1177/0047287515625129

[B95] Zhao F. (2014). A holistic and integrated approach to theorizing strategic alliances of small and medium-sized enterprises. Bus. Process Manag. J. 20, 887–905. 10.1108/BPMJ-01-2013-0004

[B96] Ziakas V. Costa C. (2011). Event portfolio and multi-purpose development: establishing the conceptual grounds. SPORT Manag. Rev. 14, 409–423. 10.1016/j.smr.2010.09.003

